# 1-Phenyl-3-{4-[4-(4-undecyl­oxybenzoyl­oxy)phenyl­oxycarbon­yl]phen­yl}triazene 1-oxide

**DOI:** 10.1107/S1600536808005904

**Published:** 2008-03-07

**Authors:** Purak Das, Achintesh Narayan Biswas, Shailesh Upreti, Pradip Kumar Mandal, Pinaki Bandyopadhyay

**Affiliations:** a Department of Chemistry, University of North Bengal, Siliguri 734 013, India; b Department of Chemistry, Indian Institute of Technology, Delhi, New Delhi 110 016, India; c Department of Physics, University of North Bengal, Siliguri 734 013, India

## Abstract

The X-ray crystallographic study of the title compound, C_37_H_41_N_3_O_6_, at 150 K establishes the *N*-oxide form of the triazene 1-oxide unit. There is one intra­molecular N—H⋯O hydrogen-bonding inter­action and the crystal packing is stabilized by one N—H⋯O, three C—H⋯O and three C—H⋯π inter­molecular inter­actions. The dihedral angles between pairs of adjacent benzene rings are 14.9 (3), 56.3 (1) and 56.0 (1)°

## Related literature

For related literature, see: Ciunik *et al.* (2002 ▶); Das *et al.* (2005 ▶); Hörner *et al.* (2002 ▶); Rapta *et al.* (1996 ▶); Samanta *et al.* (1997 ▶); Vaughan *et al.* (1992 ▶); Wilman (1988 ▶).
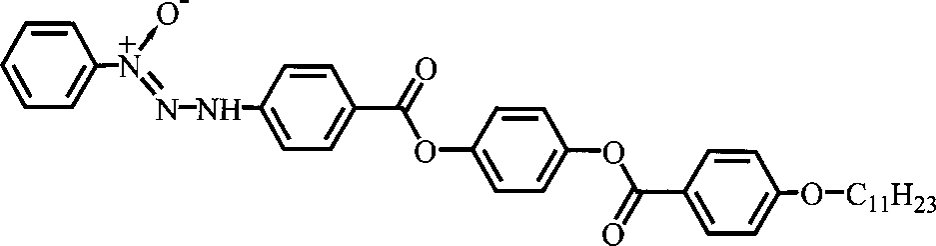


## Experimental

### 

#### Crystal data

C_37_H_41_N_3_O_6_
*M* *_r_* = 623.73
Triclinic, 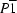

*a* = 5.674 (3) Å
*b* = 12.039 (7) Å
*c* = 24.931 (15) Å
α = 101.779 (10)°
β = 92.826 (11)°
γ = 96.565 (10)°
*V* = 1651.6 (17) Å^3^
*Z* = 2
Mo *K*α radiation
μ = 0.09 mm^−1^
*T* = 150 (2) K
0.33 × 0.09 × 0.04 mm

#### Data collection

Bruker SMART APEX CCD area detector diffractometer
Absorption correction: multi-scan (*SADABS*; Sheldrick, 1996 ▶) *T*_min_ = 0.988, *T*_max_ = 0.995
11715 measured reflections
5812 independent reflections
3464 reflections with *I* > 2σ(*I*)
*R*_int_ = 0.072

#### Refinement

*R*[*F* ^2^ > 2σ(*F* ^2^)] = 0.096
*wR*(*F* ^2^) = 0.212
*S* = 1.08
5812 reflections
420 parameters
H atoms treated by a mixture of independent and constrained refinement
Δρ_max_ = 0.31 e Å^−3^
Δρ_min_ = −0.31 e Å^−3^

### 

Data collection: *SMART* (Bruker, 1998 ▶); cell refinement: *SAINT* (Bruker, 2000 ▶); data reduction: *SAINT*; program(s) used to solve structure: *SHELXS97* (Sheldrick, 2008 ▶); program(s) used to refine structure: *SHELXL97* (Sheldrick, 2008 ▶); molecular graphics: *SHELXTL* (Sheldrick, 2008 ▶); software used to prepare material for publication: *SHELXTL*.

## Supplementary Material

Crystal structure: contains datablocks I, global. DOI: 10.1107/S1600536808005904/fj2103sup1.cif

Structure factors: contains datablocks I. DOI: 10.1107/S1600536808005904/fj2103Isup2.hkl

Additional supplementary materials:  crystallographic information; 3D view; checkCIF report

## Figures and Tables

**Table 1:** Hydrogen-bond geometry (Å, °)

*D*—H⋯*A*	*D*—H	H⋯*A*	*D*⋯*A*	*D*—H⋯*A*
N3—H3*A*⋯O1	0.88 (5)	2.16 (5)	2.501 (5)	103 (4)
N3—H3*A*⋯O1^i^	0.88 (5)	2.10 (5)	2.909 (5)	153 (5)
C12—H12⋯O1^i^	0.95	2.44	3.225 (6)	140
C16—H16⋯O5^ii^	0.95	2.51	3.436 (5)	166
C19—H19⋯O2^iii^	0.95	2.37	3.260 (5)	157
C4—H4⋯*Cg*3^iv^	0.95	2.71	3.486 (5)	139
C15—H15⋯*Cg*1^v^	0.95	2.60	3.342 (5)	135
C28—H28*A*⋯*Cg*3^v^	0.99	2.69	3.642 (5)	161

Symmetry codes: (i) 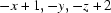; (ii) 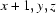; (iii) 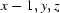; (iv) 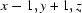; (v) 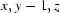. *Cg*1 and *Cg*3 are the centroids of the C1–C6 and C14–C19 rings, respectively).

**Fig. 1.**
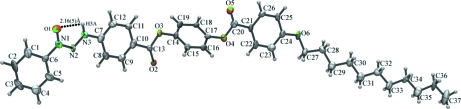
The asymmetric unit of (I), with displacement ellipsoids drawn at the 75% probability level. Dotted lines indicate the intramolecular N–H···O interaction.

**Fig. 2.**
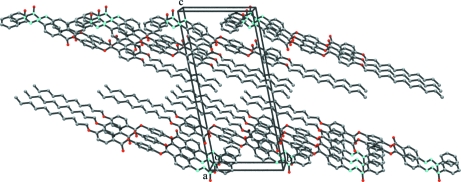
The molecular packing of (I) showing the arrangements of the molecules in the bc-plane.

**Fig. 3.**
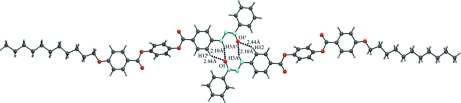
Dimerization of two molecules (I)through intermolecular N–H···O and C–H···O hydrogen bonding interactions shown by dotted lines (Symmetry code: (i) -x + 1, -y, -z + 2).

**Fig. 4.**
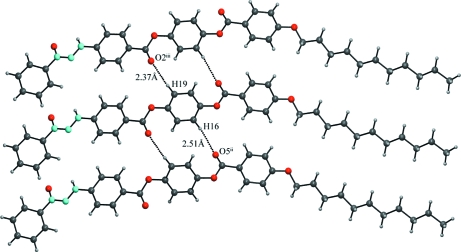
Parallel arrangement of (I) through intermolecular C–H···O interactions shown by dotted lines (Symmetry codes: (ii) x + 1, y, z; (iii) x - 1, y, z).

**Fig. 5.**
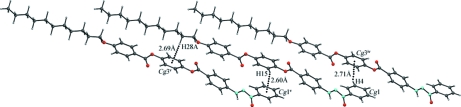
Parallel arrangement of (I) through intermolecular C—H···π interactions shown by dotted lines (Symmetry codes: (iv) x - 1, y + 1, z; (v) x, y - 1, z. Cg1 and Cg3 are the centroids of the C1–C6 and C14–C19 rings, respectively).

## supplementary crystallographic information

### Comment

Triazene-1-oxides are well known for their chelating ability (Ciunik *et al*., 2002 & Hörner *et al.*, 2002), antitumour activity (Wilman, 1988) and also for initiating radical polymerization (Rapta *et al.*, 1996). In this communication the molecular structure of a triazene-1-oxide derivative has been reported. The molecular structure of the title compound, (I), has been shown in Figure 1, with the atom-numbering scheme. The planar phenyl moiety and trigonal planar geometry of the triazene N3 atom strongly suggest a resonance interaction extending over the C6, N1, N2 and N3 atoms. The N1—N2 and N2—N3 distances are in good agreement with the reported values of other triazene-1-oxides (Samanta *et al.*, 1997; Vaughan *et al.*, 1992). The shorter length of N1—N2 indicates its double-bond character and the longer N2—N3 distance is still shorter than a pure single-bond. The deviation of O1 from the molecular plane causes conjugation between N1—C6 to be less effective and is reflected in the longer N1—C6 than N3—C7 distance. There is an intramolecular N–H···O interaction within the triazene-1-oxide moiety (Figure 1, Table 1). The intramolecular hydrogen bondings result almost planar conformation of the triazene fragment of the molecule. The molecular packing of (I) has been shown in Figure 2. The intermolecular hydrogen bonding causes dimer formation of (I) (Figure 3). The crystal packing is stabilized by intermolecular one N–H···O (Figure 3, Table 1), three C–H···O (Figure 3, Figure 4, Table 1)and three C–H···π (Figure 5, Table 1) interactions (Das *et al.*, 2005). The intermolecular hydrogen bonding and intermolecular C—H··· π interactions makes the phenyltriazene-1-oxide fragments of (I) in layer arrangement (Figure 2) in the molecular assembly.

### Experimental

The title compound has been synthesized from nitobenzene, *p*-aminobenzoic acid, hydroquinone, *p*-hydroxybenzoic acid and *n*-bromoundecane using standard coupling processes involving multiple steps. The final product was crystallized by slow diffusion of ethanol into the dichloromethane solution of the title compound to yield crystals suitable for *x*-ray crystallography.

### Refinement

The N-bound H atom was located in a difference Fourier map and its coordinates and isotropic displacement parameter were freely refined. C-bound H atoms were included at calculated positions as riding atoms with C–H set to 0.95 Å for (aromatic), 0.98 Å for (CH_3_) and 0.99 Å for (CH_2_) H atoms, with *U*_iso_(H) = 1.2*U*_eq_(C) (1.5*U*_eq_ for methyl group). Some low-angle reflections were excluded from the refinement, as they were probably obscured by the beam stop.

### Crystal data

**Table:**

C_37_H_41_N_3_O_6_	*Z* = 2
*M**_r_* = 623.73	*F*_000_ = 664
Triclinic, *P*1	*D*_x_ = 1.254 Mg m^−^^3^
Hall symbol: -P 1	Mo *K*α radiation λ = 0.71073 Å
*a* = 5.674 (3) Å	Cell parameters from 5812 reflections
*b* = 12.039 (7) Å	θ = 1.7–25.5º
*c* = 24.931 (15) Å	µ = 0.09 mm^−^^1^
α = 101.779 (10)º	*T* = 150 (2) K
β = 92.826 (11)º	Needle, pale yellow
γ = 96.565 (10)º	0.33 × 0.09 × 0.04 mm
*V* = 1651.6 (17) Å^3^	

### Data collection

**Table:**

Bruker SMART APEX CCD area detector diffractometer	5812 independent reflections
Radiation source: fine-focus sealed tube	3464 reflections with *I* > 2σ(*I*)
Monochromator: graphite	*R*_int_ = 0.072
*T* = 150(2) K	θ_max_ = 25.5º
φ and ω scans	θ_min_ = 1.7º
Absorption correction: multi-scan(SADABS; Sheldrick, 1996)	*h* = −6→6
*T*_min_ = 0.988, *T*_max_ = 0.995	*k* = −14→14
11715 measured reflections	*l* = −29→30

### Refinement

**Table:**

Refinement on *F*^2^	Secondary atom site location: difference Fourier map
Least-squares matrix: full	Hydrogen site location: inferred from neighbouring sites
*R*[*F*^2^ > 2σ(*F*^2^)] = 0.096	H atoms treated by a mixture of independent and constrained refinement
*wR*(*F*^2^) = 0.212	*w* = 1/[σ^2^(*F*_o_^2^) + (0.082*P*)^2^ + 0.0843*P*] where *P* = (*F*_o_^2^ + 2*F*_c_^2^)/3
*S* = 1.08	(Δ/σ)_max_ < 0.001
5812 reflections	Δρ_max_ = 0.31 e Å^−^^3^
420 parameters	Δρ_min_ = −0.31 e Å^−^^3^
Primary atom site location: structure-invariant direct methods	Extinction correction: none

### Special details

**Table:**

Geometry. All e.s.d.'s (except the e.s.d. in the dihedral angle between two l.s. planes) are estimated using the full covariance matrix. The cell e.s.d.'s are taken into account individually in the estimation of e.s.d.'s in distances, angles and torsion angles; correlations between e.s.d.'s in cell parameters are only used when they are defined by crystal symmetry. An approximate (isotropic) treatment of cell e.s.d.'s is used for estimating e.s.d.'s involving l.s. planes.
Refinement. Refinement of *F*^2^ against ALL reflections. The weighted *R*-factor *wR* and goodness of fit *S* are based on *F*^2^, conventional *R*-factors *R* are based on *F*, with *F* set to zero for negative *F*^2^. The threshold expression of *F*^2^ > σ(*F*^2^) is used only for calculating *R*-factors(gt) *etc*. and is not relevant to the choice of reflections for refinement. *R*-factors based on *F*^2^ are statistically about twice as large as those based on *F*, and *R*- factors based on ALL data will be even larger.

### Fractional atomic coordinates and isotropic or equivalent isotropic displacement parameters (Å^2^)

**Table:**

	*x*	*y*	*z*	*U*_iso_*/*U*_eq_	
H3A	0.607 (9)	0.051 (4)	0.963 (2)	0.052 (16)*	
O1	0.6978 (5)	−0.0912 (2)	0.99661 (11)	0.0257 (7)	
O2	0.9170 (5)	0.4217 (2)	0.78920 (11)	0.0230 (7)	
O3	0.5858 (5)	0.4763 (2)	0.82795 (11)	0.0214 (7)	
O4	0.5943 (5)	0.8656 (2)	0.74197 (12)	0.0266 (8)	
O5	0.3127 (6)	0.9292 (2)	0.79658 (13)	0.0349 (9)	
O6	0.5596 (5)	1.3488 (2)	0.68317 (11)	0.0246 (7)	
N1	0.8297 (6)	−0.0997 (3)	0.95643 (14)	0.0217 (9)	
N2	0.8428 (6)	−0.0295 (3)	0.92334 (13)	0.0205 (8)	
N3	0.6991 (7)	0.0500 (3)	0.93561 (15)	0.0221 (9)	
C1	0.9117 (9)	−0.2881 (4)	0.96356 (18)	0.0310 (12)	
H1	0.7735	−0.2954	0.9832	0.037*	
C2	1.0507 (10)	−0.3763 (4)	0.9521 (2)	0.0403 (14)	
H2	1.0060	−0.4459	0.9634	0.048*	
C3	1.2551 (9)	−0.3635 (4)	0.92428 (19)	0.0358 (12)	
H3	1.3520	−0.4235	0.9175	0.043*	
C4	1.3173 (8)	−0.2634 (4)	0.90638 (17)	0.0297 (11)	
H4	1.4547	−0.2557	0.8865	0.036*	
C5	1.1826 (8)	−0.1756 (4)	0.91715 (17)	0.0257 (11)	
H5	1.2266	−0.1063	0.9054	0.031*	
C6	0.9815 (7)	−0.1889 (3)	0.94531 (16)	0.0185 (10)	
C7	0.7176 (8)	0.1385 (3)	0.90689 (16)	0.0227 (11)	
C8	0.8969 (8)	0.1530 (4)	0.87256 (18)	0.0289 (11)	
H8	1.0138	0.1019	0.8678	0.035*	
C9	0.9056 (8)	0.2408 (3)	0.84549 (18)	0.0266 (11)	
H9	1.0287	0.2494	0.8216	0.032*	
C10	0.7403 (8)	0.3177 (3)	0.85173 (16)	0.0233 (10)	
C11	0.5577 (8)	0.3021 (3)	0.88585 (17)	0.0241 (11)	
H11	0.4395	0.3525	0.8901	0.029*	
C12	0.5485 (8)	0.2143 (4)	0.91320 (17)	0.0263 (11)	
H12	0.4249	0.2050	0.9369	0.032*	
C13	0.7644 (7)	0.4083 (3)	0.81940 (16)	0.0167 (9)	
C14	0.5992 (7)	0.5729 (3)	0.80380 (16)	0.0172 (9)	
C15	0.7954 (7)	0.6542 (3)	0.81402 (16)	0.0176 (9)	
H15	0.9327	0.6432	0.8347	0.021*	
C16	0.7896 (7)	0.7526 (3)	0.79363 (16)	0.0168 (10)	
H16	0.9236	0.8099	0.8000	0.020*	
C17	0.5872 (7)	0.7667 (3)	0.76399 (17)	0.0188 (10)	
C18	0.3911 (7)	0.6858 (3)	0.75446 (17)	0.0190 (10)	
H18	0.2526	0.6974	0.7344	0.023*	
C19	0.3974 (7)	0.5858 (3)	0.77461 (16)	0.0198 (10)	
H19	0.2642	0.5280	0.7681	0.024*	
C20	0.4459 (8)	0.9433 (3)	0.76209 (17)	0.0239 (10)	
C21	0.4854 (7)	1.0462 (3)	0.73795 (16)	0.0177 (10)	
C22	0.6686 (8)	1.0670 (3)	0.70587 (18)	0.0277 (11)	
H22	0.7747	1.0115	0.6968	0.033*	
C23	0.7018 (8)	1.1675 (4)	0.68634 (17)	0.0273 (11)	
H23	0.8302	1.1810	0.6645	0.033*	
C24	0.5457 (8)	1.2473 (3)	0.69914 (17)	0.0219 (10)	
C25	0.3546 (8)	1.2251 (3)	0.72996 (17)	0.0226 (10)	
H25	0.2453	1.2793	0.7381	0.027*	
C26	0.3238 (8)	1.1258 (3)	0.74858 (17)	0.0226 (10)	
H26	0.1911	1.1106	0.7690	0.027*	
C27	0.7669 (8)	1.3862 (3)	0.65780 (18)	0.0249 (11)	
H27A	0.7710	1.3379	0.6207	0.030*	
H27B	0.9127	1.3809	0.6801	0.030*	
C28	0.7526 (8)	1.5068 (3)	0.65407 (18)	0.0261 (11)	
H28A	0.7516	1.5531	0.6917	0.031*	
H28B	0.5992	1.5103	0.6342	0.031*	
C29	0.9515 (8)	1.5615 (3)	0.62570 (18)	0.0275 (11)	
H29A	0.9433	1.5217	0.5866	0.033*	
H29B	1.1071	1.5531	0.6432	0.033*	
C30	0.9318 (8)	1.6876 (3)	0.62928 (18)	0.0287 (11)	
H30A	0.9462	1.7262	0.6686	0.034*	
H30B	0.7706	1.6944	0.6142	0.034*	
C31	1.1121 (9)	1.7520 (4)	0.5999 (2)	0.0328 (12)	
H31A	1.2744	1.7488	0.6155	0.039*	
H31B	1.1010	1.7141	0.5605	0.039*	
C32	1.0726 (8)	1.8755 (4)	0.60495 (18)	0.0291 (11)	
H32A	1.0965	1.9136	0.6443	0.035*	
H32B	0.9045	1.8774	0.5929	0.035*	
C33	1.2298 (9)	1.9452 (3)	0.57279 (18)	0.0304 (12)	
H33A	1.3978	1.9488	0.5865	0.037*	
H33B	1.2138	1.9056	0.5336	0.037*	
C34	1.1690 (8)	2.0658 (4)	0.57715 (18)	0.0286 (11)	
H34A	1.1843	2.1048	0.6164	0.034*	
H34B	1.0006	2.0617	0.5636	0.034*	
C35	1.3232 (9)	2.1382 (4)	0.54541 (18)	0.0331 (12)	
H35A	1.4912	2.1447	0.5597	0.040*	
H35B	1.3117	2.0985	0.5063	0.040*	
C36	1.2542 (9)	2.2568 (3)	0.5491 (2)	0.0367 (13)	
H36A	1.2691	2.2969	0.5882	0.044*	
H36B	1.0850	2.2501	0.5356	0.044*	
C37	1.4037 (10)	2.3293 (4)	0.5165 (2)	0.0529 (16)	
H37A	1.3981	2.2882	0.4781	0.079*	
H37B	1.5688	2.3439	0.5322	0.079*	
H37C	1.3402	2.4021	0.5181	0.079*	

### Atomic displacement parameters (Å^2^)

**Table:**

	*U*^11^	*U*^22^	*U*^33^	*U*^12^	*U*^13^	*U*^23^
O1	0.0294 (19)	0.0308 (18)	0.0201 (16)	0.0072 (15)	0.0124 (14)	0.0086 (14)
O2	0.0202 (18)	0.0129 (15)	0.0377 (18)	0.0026 (13)	0.0121 (14)	0.0070 (13)
O3	0.0252 (18)	0.0126 (15)	0.0298 (17)	0.0039 (13)	0.0115 (14)	0.0098 (13)
O4	0.0294 (19)	0.0198 (16)	0.0383 (18)	0.0104 (14)	0.0163 (15)	0.0166 (14)
O5	0.033 (2)	0.0234 (17)	0.056 (2)	0.0066 (15)	0.0228 (17)	0.0189 (16)
O6	0.0303 (19)	0.0099 (15)	0.0358 (17)	−0.0013 (13)	0.0065 (15)	0.0115 (13)
N1	0.023 (2)	0.0153 (19)	0.025 (2)	0.0037 (16)	0.0046 (17)	−0.0024 (16)
N2	0.027 (2)	0.0101 (18)	0.027 (2)	0.0056 (16)	0.0115 (17)	0.0065 (16)
N3	0.025 (2)	0.020 (2)	0.025 (2)	0.0096 (17)	0.0082 (18)	0.0086 (17)
C1	0.045 (3)	0.022 (3)	0.029 (3)	0.010 (2)	0.007 (2)	0.008 (2)
C2	0.061 (4)	0.027 (3)	0.049 (3)	0.029 (3)	0.018 (3)	0.028 (2)
C3	0.035 (3)	0.032 (3)	0.043 (3)	0.021 (2)	0.000 (2)	0.006 (2)
C4	0.029 (3)	0.028 (3)	0.028 (3)	0.003 (2)	0.002 (2)	−0.003 (2)
C5	0.021 (3)	0.022 (2)	0.029 (2)	0.003 (2)	0.001 (2)	−0.007 (2)
C6	0.015 (2)	0.015 (2)	0.024 (2)	−0.0011 (18)	0.0017 (19)	0.0015 (18)
C7	0.030 (3)	0.016 (2)	0.019 (2)	−0.004 (2)	0.004 (2)	−0.0008 (19)
C8	0.026 (3)	0.023 (2)	0.045 (3)	0.011 (2)	0.009 (2)	0.018 (2)
C9	0.024 (3)	0.025 (3)	0.035 (3)	0.004 (2)	0.013 (2)	0.012 (2)
C10	0.027 (3)	0.017 (2)	0.023 (2)	−0.005 (2)	0.000 (2)	0.0015 (19)
C11	0.021 (3)	0.022 (2)	0.035 (3)	0.009 (2)	0.014 (2)	0.013 (2)
C12	0.029 (3)	0.030 (3)	0.024 (2)	0.008 (2)	0.012 (2)	0.012 (2)
C13	0.015 (2)	0.011 (2)	0.022 (2)	0.0009 (18)	0.0038 (19)	−0.0020 (18)
C14	0.018 (2)	0.010 (2)	0.024 (2)	−0.0005 (18)	0.0022 (19)	0.0087 (18)
C15	0.014 (2)	0.015 (2)	0.024 (2)	0.0005 (18)	−0.0001 (18)	0.0059 (18)
C16	0.006 (2)	0.011 (2)	0.030 (2)	−0.0058 (17)	0.0013 (18)	0.0000 (18)
C17	0.014 (2)	0.016 (2)	0.027 (2)	0.0025 (18)	0.0104 (19)	0.0034 (19)
C18	0.010 (2)	0.015 (2)	0.033 (2)	0.0012 (18)	0.0071 (19)	0.0068 (19)
C19	0.014 (2)	0.008 (2)	0.034 (2)	−0.0077 (17)	0.004 (2)	−0.0001 (19)
C20	0.022 (3)	0.021 (2)	0.028 (2)	0.001 (2)	0.003 (2)	0.006 (2)
C21	0.016 (2)	0.0035 (19)	0.032 (2)	−0.0044 (17)	−0.0011 (19)	0.0044 (18)
C22	0.031 (3)	0.014 (2)	0.041 (3)	0.002 (2)	0.010 (2)	0.011 (2)
C23	0.025 (3)	0.028 (3)	0.029 (3)	0.003 (2)	0.008 (2)	0.004 (2)
C24	0.025 (3)	0.016 (2)	0.026 (2)	0.000 (2)	0.008 (2)	0.0074 (19)
C25	0.021 (3)	0.010 (2)	0.035 (3)	0.0000 (19)	0.002 (2)	0.0002 (19)
C26	0.017 (2)	0.020 (2)	0.030 (2)	−0.0018 (19)	0.009 (2)	0.005 (2)
C27	0.032 (3)	0.015 (2)	0.029 (2)	0.000 (2)	0.012 (2)	0.0089 (19)
C28	0.027 (3)	0.022 (2)	0.031 (3)	0.007 (2)	0.010 (2)	0.006 (2)
C29	0.032 (3)	0.026 (3)	0.025 (2)	0.001 (2)	0.004 (2)	0.007 (2)
C30	0.029 (3)	0.022 (3)	0.037 (3)	0.002 (2)	0.005 (2)	0.012 (2)
C31	0.034 (3)	0.027 (3)	0.041 (3)	0.005 (2)	0.007 (2)	0.012 (2)
C32	0.029 (3)	0.032 (3)	0.028 (2)	0.003 (2)	0.010 (2)	0.011 (2)
C33	0.037 (3)	0.020 (2)	0.033 (3)	−0.006 (2)	0.008 (2)	0.005 (2)
C34	0.018 (3)	0.030 (3)	0.036 (3)	−0.010 (2)	−0.003 (2)	0.010 (2)
C35	0.041 (3)	0.030 (3)	0.029 (3)	−0.002 (2)	0.005 (2)	0.011 (2)
C36	0.046 (3)	0.015 (2)	0.046 (3)	−0.013 (2)	−0.004 (3)	0.009 (2)
C37	0.073 (4)	0.031 (3)	0.055 (4)	−0.013 (3)	−0.011 (3)	0.024 (3)

### Geometric parameters (Å, °)

**Table:**

O1—N1	1.274 (4)	C19—H19	0.9500
O2—C13	1.193 (4)	C20—C21	1.484 (6)
O3—C13	1.372 (5)	C21—C22	1.373 (5)
O3—C14	1.410 (4)	C21—C26	1.397 (6)
O4—C20	1.366 (5)	C22—C23	1.391 (6)
O4—C17	1.407 (5)	C22—H22	0.9500
O5—C20	1.197 (5)	C23—C24	1.381 (6)
O6—C24	1.356 (5)	C23—H23	0.9500
O6—C27	1.433 (5)	C24—C25	1.392 (5)
N1—N2	1.295 (4)	C25—C26	1.365 (6)
N1—C6	1.444 (5)	C25—H25	0.9500
N2—N3	1.326 (5)	C26—H26	0.9500
N3—C7	1.397 (5)	C27—C28	1.485 (6)
N3—H3A	0.89 (5)	C27—H27A	0.9900
C1—C2	1.385 (6)	C27—H27B	0.9900
C1—C6	1.386 (6)	C28—C29	1.518 (6)
C1—H1	0.9500	C28—H28A	0.9900
C2—C3	1.390 (6)	C28—H28B	0.9900
C2—H2	0.9500	C29—C30	1.520 (6)
C3—C4	1.384 (6)	C29—H29A	0.9900
C3—H3	0.9500	C29—H29B	0.9900
C4—C5	1.365 (6)	C30—C31	1.520 (6)
C4—H4	0.9500	C30—H30A	0.9900
C5—C6	1.379 (5)	C30—H30B	0.9900
C5—H5	0.9500	C31—C32	1.509 (6)
C7—C8	1.380 (6)	C31—H31A	0.9900
C7—C12	1.391 (6)	C31—H31B	0.9900
C8—C9	1.363 (6)	C32—C33	1.522 (5)
C8—H8	0.9500	C32—H32A	0.9900
C9—C10	1.384 (6)	C32—H32B	0.9900
C9—H9	0.9500	C33—C34	1.514 (6)
C10—C11	1.393 (5)	C33—H33A	0.9900
C10—C13	1.482 (6)	C33—H33B	0.9900
C11—C12	1.368 (6)	C34—C35	1.525 (6)
C11—H11	0.9500	C34—H34A	0.9900
C12—H12	0.9500	C34—H34B	0.9900
C14—C19	1.367 (6)	C35—C36	1.510 (6)
C14—C15	1.373 (6)	C35—H35A	0.9900
C15—C16	1.384 (5)	C35—H35B	0.9900
C15—H15	0.9500	C36—C37	1.527 (6)
C16—C17	1.381 (6)	C36—H36A	0.9900
C16—H16	0.9500	C36—H36B	0.9900
C17—C18	1.369 (6)	C37—H37A	0.9800
C18—C19	1.399 (5)	C37—H37B	0.9800
C18—H18	0.9500	C37—H37C	0.9800
			
C13—O3—C14	117.5 (3)	C24—C23—H23	120.4
C20—O4—C17	117.0 (3)	C22—C23—H23	120.4
C24—O6—C27	119.1 (3)	O6—C24—C23	125.0 (4)
O1—N1—N2	123.7 (3)	O6—C24—C25	115.1 (4)
O1—N1—C6	121.5 (3)	C23—C24—C25	119.9 (4)
N2—N1—C6	114.9 (3)	C26—C25—C24	120.2 (4)
N1—N2—N3	111.9 (3)	C26—C25—H25	119.9
N2—N3—C7	117.2 (4)	C24—C25—H25	119.9
N2—N3—H3A	121 (3)	C25—C26—C21	120.6 (4)
C7—N3—H3A	122 (3)	C25—C26—H26	119.7
C2—C1—C6	117.6 (4)	C21—C26—H26	119.7
C2—C1—H1	121.2	O6—C27—C28	107.1 (3)
C6—C1—H1	121.2	O6—C27—H27A	110.3
C1—C2—C3	120.5 (4)	C28—C27—H27A	110.3
C1—C2—H2	119.7	O6—C27—H27B	110.3
C3—C2—H2	119.7	C28—C27—H27B	110.3
C4—C3—C2	119.9 (4)	H27A—C27—H27B	108.6
C4—C3—H3	120.0	C27—C28—C29	115.6 (4)
C2—C3—H3	120.0	C27—C28—H28A	108.4
C5—C4—C3	120.4 (4)	C29—C28—H28A	108.4
C5—C4—H4	119.8	C27—C28—H28B	108.4
C3—C4—H4	119.8	C29—C28—H28B	108.4
C4—C5—C6	119.0 (4)	H28A—C28—H28B	107.4
C4—C5—H5	120.5	C28—C29—C30	110.8 (4)
C6—C5—H5	120.5	C28—C29—H29A	109.5
C5—C6—C1	122.4 (4)	C30—C29—H29A	109.5
C5—C6—N1	121.1 (4)	C28—C29—H29B	109.5
C1—C6—N1	116.5 (4)	C30—C29—H29B	109.5
C8—C7—C12	119.1 (4)	H29A—C29—H29B	108.1
C8—C7—N3	122.3 (4)	C29—C30—C31	116.6 (4)
C12—C7—N3	118.6 (4)	C29—C30—H30A	108.1
C9—C8—C7	119.7 (4)	C31—C30—H30A	108.1
C9—C8—H8	120.1	C29—C30—H30B	108.1
C7—C8—H8	120.1	C31—C30—H30B	108.1
C8—C9—C10	122.0 (4)	H30A—C30—H30B	107.3
C8—C9—H9	119.0	C32—C31—C30	112.1 (4)
C10—C9—H9	119.0	C32—C31—H31A	109.2
C9—C10—C11	118.3 (4)	C30—C31—H31A	109.2
C9—C10—C13	117.2 (4)	C32—C31—H31B	109.2
C11—C10—C13	124.4 (4)	C30—C31—H31B	109.2
C12—C11—C10	120.0 (4)	H31A—C31—H31B	107.9
C12—C11—H11	120.0	C31—C32—C33	116.4 (4)
C10—C11—H11	120.0	C31—C32—H32A	108.2
C11—C12—C7	121.0 (4)	C33—C32—H32A	108.2
C11—C12—H12	119.5	C31—C32—H32B	108.2
C7—C12—H12	119.5	C33—C32—H32B	108.2
O2—C13—O3	123.2 (4)	H32A—C32—H32B	107.3
O2—C13—C10	125.4 (4)	C34—C33—C32	113.2 (4)
O3—C13—C10	111.4 (3)	C34—C33—H33A	108.9
C19—C14—C15	122.5 (4)	C32—C33—H33A	108.9
C19—C14—O3	115.9 (3)	C34—C33—H33B	108.9
C15—C14—O3	121.3 (4)	C32—C33—H33B	108.9
C14—C15—C16	118.8 (4)	H33A—C33—H33B	107.7
C14—C15—H15	120.6	C33—C34—C35	114.7 (4)
C16—C15—H15	120.6	C33—C34—H34A	108.6
C17—C16—C15	119.4 (4)	C35—C34—H34A	108.6
C17—C16—H16	120.3	C33—C34—H34B	108.6
C15—C16—H16	120.3	C35—C34—H34B	108.6
C18—C17—C16	121.6 (4)	H34A—C34—H34B	107.6
C18—C17—O4	121.3 (4)	C36—C35—C34	113.5 (4)
C16—C17—O4	117.1 (4)	C36—C35—H35A	108.9
C17—C18—C19	119.2 (4)	C34—C35—H35A	108.9
C17—C18—H18	120.4	C36—C35—H35B	108.9
C19—C18—H18	120.4	C34—C35—H35B	108.9
C14—C19—C18	118.6 (4)	H35A—C35—H35B	107.7
C14—C19—H19	120.7	C35—C36—C37	114.0 (4)
C18—C19—H19	120.7	C35—C36—H36A	108.7
O5—C20—O4	122.6 (4)	C37—C36—H36A	108.7
O5—C20—C21	126.5 (4)	C35—C36—H36B	108.7
O4—C20—C21	110.8 (3)	C37—C36—H36B	108.7
C22—C21—C26	118.6 (4)	H36A—C36—H36B	107.6
C22—C21—C20	124.1 (4)	C36—C37—H37A	109.5
C26—C21—C20	117.2 (4)	C36—C37—H37B	109.5
C21—C22—C23	121.3 (4)	H37A—C37—H37B	109.5
C21—C22—H22	119.3	C36—C37—H37C	109.5
C23—C22—H22	119.3	H37A—C37—H37C	109.5
C24—C23—C22	119.1 (4)	H37B—C37—H37C	109.5
			
O1—N1—N2—N3	−1.5 (5)	C15—C16—C17—C18	0.3 (6)
C6—N1—N2—N3	179.8 (3)	C15—C16—C17—O4	−177.1 (3)
N1—N2—N3—C7	172.8 (3)	C20—O4—C17—C18	67.9 (5)
C6—C1—C2—C3	1.2 (7)	C20—O4—C17—C16	−114.7 (4)
C1—C2—C3—C4	−1.8 (8)	C16—C17—C18—C19	−0.9 (6)
C2—C3—C4—C5	1.7 (7)	O4—C17—C18—C19	176.4 (3)
C3—C4—C5—C6	−1.1 (7)	C15—C14—C19—C18	0.0 (6)
C4—C5—C6—C1	0.5 (7)	O3—C14—C19—C18	173.5 (3)
C4—C5—C6—N1	−178.3 (4)	C17—C18—C19—C14	0.7 (6)
C2—C1—C6—C5	−0.6 (7)	C17—O4—C20—O5	−0.6 (6)
C2—C1—C6—N1	178.3 (4)	C17—O4—C20—C21	176.0 (3)
O1—N1—C6—C5	−155.3 (4)	O5—C20—C21—C22	168.0 (5)
N2—N1—C6—C5	23.4 (6)	O4—C20—C21—C22	−8.5 (6)
O1—N1—C6—C1	25.8 (6)	O5—C20—C21—C26	−12.5 (7)
N2—N1—C6—C1	−155.5 (4)	O4—C20—C21—C26	171.1 (4)
N2—N3—C7—C8	−8.6 (6)	C26—C21—C22—C23	3.2 (6)
N2—N3—C7—C12	171.3 (4)	C20—C21—C22—C23	−177.3 (4)
C12—C7—C8—C9	0.0 (7)	C21—C22—C23—C24	−0.6 (7)
N3—C7—C8—C9	179.9 (4)	C27—O6—C24—C23	−9.4 (6)
C7—C8—C9—C10	0.7 (7)	C27—O6—C24—C25	171.6 (4)
C8—C9—C10—C11	−1.5 (7)	C22—C23—C24—O6	179.3 (4)
C8—C9—C10—C13	−178.8 (4)	C22—C23—C24—C25	−1.7 (7)
C9—C10—C11—C12	1.6 (6)	O6—C24—C25—C26	−179.6 (4)
C13—C10—C11—C12	178.7 (4)	C23—C24—C25—C26	1.4 (6)
C10—C11—C12—C7	−1.0 (7)	C24—C25—C26—C21	1.2 (6)
C8—C7—C12—C11	0.2 (7)	C22—C21—C26—C25	−3.5 (6)
N3—C7—C12—C11	−179.7 (4)	C20—C21—C26—C25	176.9 (4)
C14—O3—C13—O2	−6.4 (5)	C24—O6—C27—C28	−170.1 (4)
C14—O3—C13—C10	174.1 (3)	O6—C27—C28—C29	−177.2 (4)
C9—C10—C13—O2	−1.4 (6)	C27—C28—C29—C30	−174.4 (4)
C11—C10—C13—O2	−178.5 (4)	C28—C29—C30—C31	−176.8 (4)
C9—C10—C13—O3	178.0 (4)	C29—C30—C31—C32	178.6 (4)
C11—C10—C13—O3	0.9 (6)	C30—C31—C32—C33	−174.8 (4)
C13—O3—C14—C19	130.4 (4)	C31—C32—C33—C34	176.4 (4)
C13—O3—C14—C15	−56.0 (5)	C32—C33—C34—C35	180.0 (4)
C19—C14—C15—C16	−0.6 (6)	C33—C34—C35—C36	178.3 (4)
O3—C14—C15—C16	−173.7 (3)	C34—C35—C36—C37	−178.7 (4)
C14—C15—C16—C17	0.4 (6)		

### Hydrogen-bond geometry (Å, °)

**Table:**

*D*—H···*A*	*D*—H	H···*A*	*D*···*A*	*D*—H···*A*
N3—H3A···O1	0.88 (5)	2.16 (5)	2.501 (5)	103 (4)
N3—H3A···O1^i^	0.88 (5)	2.10 (5)	2.909 (5)	153 (5)
C12—H12···O1^i^	0.95	2.44	3.225 (6)	140
C16—H16···O5^ii^	0.95	2.51	3.436 (5)	166
C19—H19···O2^iii^	0.95	2.37	3.260 (5)	157
C4—H4···Cg3^iv^	0.95	2.71	3.486 (5)	139
C15—H15···Cg1^v^	0.95	2.60	3.342 (5)	135
C28—H28A···Cg3^v^	0.99	2.69	3.642 (5)	161

Symmetry codes: (i) −*x*+1, −*y*, −*z*+2; (ii) *x*+1, *y*, *z*; (iii) *x*−1, *y*, *z*; (iv) *x*−1, *y*+1, *z*; (v) *x*, *y*−1, *z*.
